# Evolution of monkeypox virus from 2017 to 2022: In the light of point mutations

**DOI:** 10.3389/fmicb.2022.1037598

**Published:** 2022-12-14

**Authors:** Perumal Arumugam Desingu, T. P. Rubeni, Nagalingam R. Sundaresan

**Affiliations:** Department of Microbiology and Cell Biology, Indian Institute of Science, Bengaluru, India

**Keywords:** monkeypox, multi-country outbreak-2022, monkeypox virus evolution from 2017 to 2022, selection and mutation pressure, adaptive evolution of MPXVs in humans, point mutations

## Abstract

Monkeypox virus (MPXV) causing multi-country outbreak-2022 is related to viruses caused outbreak-2017–2018 in West Africa. Still not fully understood which proteins of the MPXV discovered in Nigeria in 2017 have mutated through different lineages to the extent that it could cause a multi-country outbreak in 2022; similarly, codon usage bias, host adaptation indices, and the role of selection or mutation pressure in the mutated genes are also not fully studied. Here we report that according to the available sequence data this monkeypox virus acquires point mutations in multiple proteins in each period, and these point mutations accumulate and become a virus that can root outbreak-2022. Viruses exported from Nigeria to Singapore, Israel, and the United Kingdom in 2018–2019 were developed as evolutionary ancestors to B.1 viruses (MPXVs causing multi-country outbreak-2022) through MPXV/United States/2021/MD virus. Although these exported viruses have different amino acid mutations in different proteins, amino acid mutations in 10 proteins are common among them. The MPXV-United Kingdom-P2 virus evolved with only mutations in these 10 proteins and further evolved into MPXV/United States/2021/MD with amino acid mutations in 26 (including amino acid mutations in 10 proteins of the MPXV-United States-P2) proteins. It is noteworthy that specific amino acid mutations in these 22/26 (presence in MPXV/United States/2021/MD) proteins are present in B.1 viruses. Further, analysis of Relative Synonymous Codon Usage (RSCU), Synonymous Codon Usage Fraction (SCUF), and Effective Number of Codons (ENc) revealed codon usage bias in genes that exhibited nucleotide mutations in lineage B.1. Also, host adaptation indices analyzes such as Codon Adaptation Index (CAI), Expected-CAI (eCAI), Relative Codon Deoptimization Index (RCDI) and Expected value for the RCDI (eRCDI) analyzes reveal that the genes that demonstrated nucleotide mutations in lineage B.1 are favorable for human adaptation. Similarly, ENc-GC3s plot, Neutrality plot, and Parity Rule 2 (PR2)-bias plot analyzes suggest a major role of selection pressure than mutation pressure in the evolution of genes displaying nucleotide mutations in lineage B.1. Overall, from 2017 to 2022, MPXV’s mutation and spread suggests that this virus continues to evolve through point mutation in the genes according to the available sequence data.

## Introduction

Human monkeypox is a zoonotic infection caused by the monkeypox virus (MPXV) in Orthopoxvirus (Adler et al., 2022) which contains a double-stranded DNA genome of ≈186–228 kb and encodes ~200 genes; however, MPXV has a genome size of ≈197 kb with nearly 190 genes (Kugelman et al., 2014; Muhlemann et al., 2020; Desingu, 2022). The central ~100,000-nt region of the genome of this virus contains conserved genes required for virus transcription and replication, and its left and right arms contain genes that determine host innate immunity and host range (Kugelman et al., 2014; Muhlemann et al., 2020). Historically, from 1958 to 1964, MPXVs were detected in monkeys in European countries such as Denmark (Von Magnus et al., 1956), Netherlands (Gispen et al., 1967; Parker and Buller, 2013), and the United States (Mcconnell et al., 1962). The MPXV virus that infects humans was first discovered in 1970 in the Congo (Ladnyj et al., 1972). Subsequently, the MPXV virus created several periodic outbreaks in Central African countries (Heymann et al., 1998; Learned et al., 2005; Formenty et al., 2010; Rimoin et al., 2010; Nolen et al., 2016). Similarly, in West African countries, the MPXV virus, which lacks human-to-human transmission and causes a mild disease, has developed short outbreaks from 1970 to 1981 (Reynolds and Damon, 2012; Rezza, 2019). Further, phylogenetic analysis divided these viruses into two separate clades (Hutin et al., 2001; Reynolds et al., 2007; Brown and Leggat, 2016; Yinka-Ogunleye et al., 2019): the central African clade of MPXVs (CA-MPXVs/Clade I), which causes severe illness in central African countries (Breman et al., 1980; Hutin et al., 2001; Reynolds et al., 2007; Brown and Leggat, 2016), and the West African clades (WA-MPXVs/Clade IIa) of MPXV which causes a mild infection in west African countries (Reynolds and Damon, 2012; Rezza, 2019). Importantly, WA-MPXV/Clade IIa viruses have become a human-to-human transmission, causing an outbreak-2017–2018 in Nigeria (Jezek et al., 1986; Rezza, 2019; Yinka-Ogunleye et al., 2019). Viruses spread from this outbreak-2017–2018 to countries such as United Kingdom (Mauldin et al., 2022), Israel (Yinka-Ogunleye et al., 2019), and Singapore (Ng et al., 2019). It is also worth noting that viruses found in countries such as the United Kingdom, Israel, and Singapore were slightly different from those that outbreaks in different parts of Nigeria except Bayelsa (Mauldin et al., 2022). A traveler from Nigeria to the United States (in 2021) has been diagnosed with monkeypox infection, and the virus was reported to be related to the virus that caused the outbreak-2017–2018 in Nigeria, in particular, it is noteworthy that the infection did not spread to healthcare workers (Costello et al., 2022).

A recent study reported that the MPXV viruses causing the multi-country outbreak-2022 are related to the viruses that caused the outbreak-2017–2018 in Nigeria (Isidro et al., 2022). Furthermore, these studies divided MPXV viruses into three distinct groups as follows: (i) CA-MPXVs as Clade-1//Clade I, (ii) WA-MPXVs detected before 2017 as Clade-2//Clade IIa, and (iii) WA-MPXVs detected after 2017 as Clade-3/Clade IIb using core SNPs (Isidro et al., 2022) and inverted terminal repeats (ITR; Happi et al., 2022). In addition, Clade-3/Clade IIb viruses are sub-divided into A.1 and A.2, and A.1 is further divided into two lineages, A.1.1 and B.1 (multi-country MPXV-2022) (Isidro et al., 2022). In particular, the microevolution of SNPs between MPXV-UK_P2 (accession MT903344.1) virus in the A.1 sub-group and viruses in the B.1 lineage has been studied (Isidro et al., 2022). In this situation, the Clade-3 viruses that acquired the character of human-to-human transmission in 2017 are still not fully understood about what kind of evolutionary development they have undergone until 2022 and which mutations have accumulated as a positive selection during the evolutionary process and evolved to the extent that is causing multi-country outbreak-2022. In this context, in this study, we find out and report what kind of evolution has been achieved in the Clade-3/Clade IIb viruses from 2017 to 2022 to cause the multi-country outbreak-2022.

## Results and discussion

**Figure 1:**
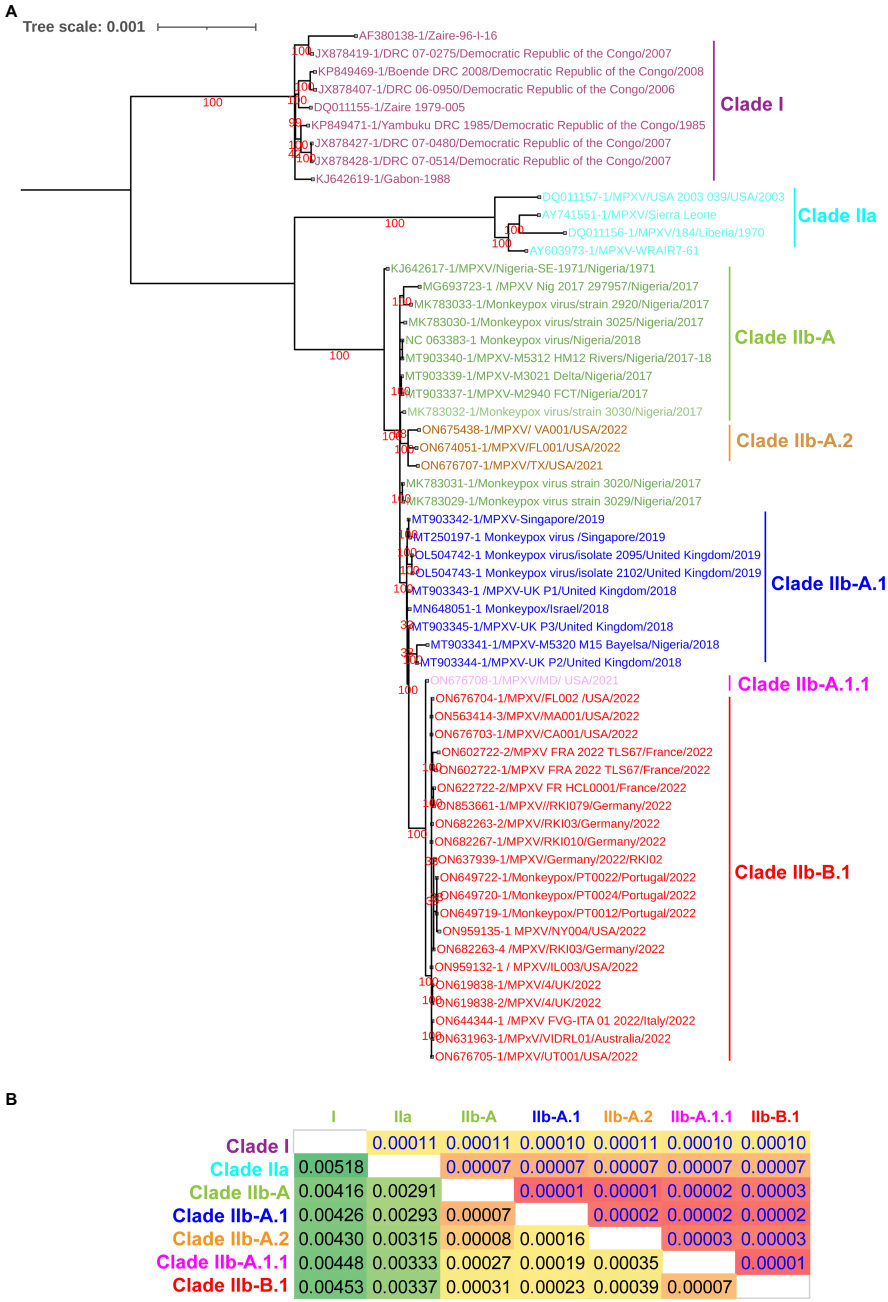
Complete genome-level genetic diversity in the different clades and lineages of the monkeypox viruses. **(A)** The MPXV different clades and lineages at whole genome levels are also classified as similar according to a recent report that classified MPXV at core SNPs (Isidro et al., 2022) andFIGURE 1 (Continued)inverted terminal repeats (ITR; Happi et al., 2022). **(B)** Complete genome levels of genetic diversity (NBGMD) among different clades and lineages MPXVs. Complete genome sequences of different clades and lineages of MPXVs in panel **A** were used for this analysis. The measured standard errors in the NBGMD analysis were displayed above the diagonal in the table.

Since a recent study classified clade IIb MPXV viruses into different lineages such as A, A.1, A.1.1, A.2, and B.1 using core SNPs (Single nucleotide polymorphisms) (Isidro et al., 2022) and ITR (Happi et al., 2022), we were interested in finding out whether these lineages could also be separated at the complete genome level. In our complete genome-wide phylogenetic analysis, we observed that clade IIb MPXV viruses split into different lineages (Figure 1A), as reported at the levels of SNPs (Isidro et al., 2022) and ITR (Happi et al., 2022). In this analysis, the viruses responsible for the outbreak in West Africa in 2017–2018 clustered together to form lineage A or hMPXV-1A (Figure 1A). Also, it is worth stating that viruses in this hMPXV-1A group are more closely related to Clade IIa than Clade I at the complete genome level (Figure 1A), as we reported in our previous study (Perumal Arumugam Desingu, 2022). Similarly, Net between group mean distance (NBGMD) analysis revealed that Clade IIb viruses shared 0.291–0.337% and 0.416–0.453% genetic diversity with Clade IIa and Clade I viruses at the complete genome level, respectively (Figure 1A).

Furthermore, it is increasingly important that WA-MPXVs/Clade IIa viruses acquired human-to-human transmission from viruses in this hMPXV-1A group (Jezek et al., 1986; Rezza, 2019; Yinka-Ogunleye et al., 2019; Perumal Arumugam Desingu, 2022). In particular, we have reported in our previous study on the evolutionary development and genetic diversity between WA-MPXVs/Clade IIa viruses that are not capable of human-to-human transmission and hMPXV-1A viruses that are capable of human-to-human transmission (Perumal Arumugam Desingu, 2022). Here, these hMPXV-1A viruses, which acquired the character of human-to-human transmission in 2017–2018, did not cause a large multi-country outbreak in 2017–2018, but 4 years later caused a multi-country outbreak, so it is necessary to find out what evolution this virus has achieved in these 4 years. To detect this evolutionary development, we subjected the complete genome sequence of viruses that were detected from 2017 to 2022 to phylogenetic and mutational analysis.

Complete genome-wide phylogenetic analysis of the present study revealed that viruses exported from Nigeria-MPXV-outbreak-2017–2018 (hMPXV-1A) to the United Kingdom (Mauldin et al., 2022), Israel (Yinka-Ogunleye et al., 2019), and Singapore (Ng et al., 2019) form clade A.1, while viruses detected from the United States in 2021–2022 form clade A.2 (Figure 1A). Also, among the A.1 and A.2 groups, it is noteworthy that the A.2 group has the closest phylogenetic relationship with the hMPXV-1A group (Figure 1A). Interestingly, phylogenetic analysis revealed that the MPXV/United States/2021/MD (A.1.1) virus detected in the United States is an evolutionary intermediate between the A.1 group and the B.1 group (multi-country outbreak-2022 causing MPXVs) (Figure 1A). Similarly, in the NBGMD analysis, MPXV/United States/2021/MD (A.1.1) virus revealed 0.007, 0.019, 0.027, and 0.035% genetic diversity with B.1, A.1, A, and A.2 lineages, respectively, at the complete genome level (Figure 1B). Also, B.1 lineage exhibited 0.007, 0.023, 0.031, and 0.039% genetic diversity with A.1.1, A.1, A, and A.2 lineages at the complete genome level, respectively (Figure 1B). Finally, the A.1 lineage exhibited 0.019, 0.023, 0.007, and 0.016% genetic diversity with the A.1.1, B.1, A, and A.2 lineages at the complete genome level, respectively (Figure 1B). From these, it appears that the MPXV/United States/2021/MD (A.1.1) virus is an evolutionary intermediate between the A.1 and the B.1 lineages. Further, it appears that viruses closely related to the ones that caused the Nigeria-MPXV-outbreak-2017–2018 caused the infection in 2022 other than endemic areas. Collectively, the phylogenetic and NBGMD analyzes revealed that the hMPXV-1A virus has gradually evolved over different periods from 2017–2018 to 2022.

**Figure 2:**
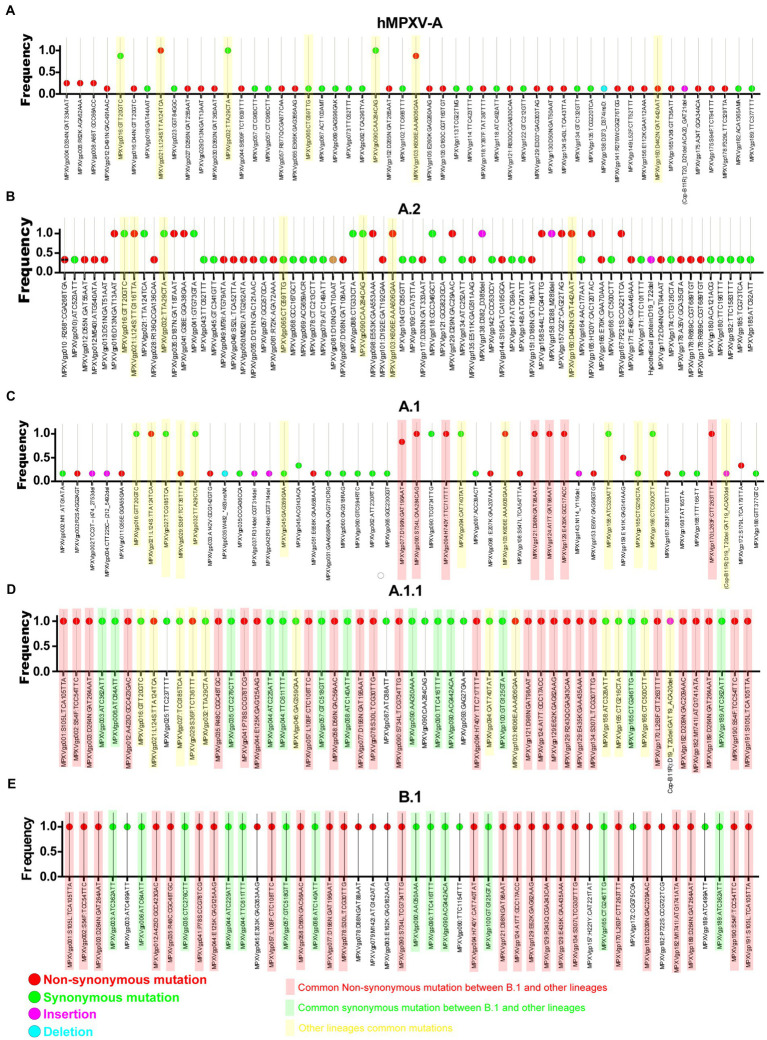
Nucleotide and amino acid mutations present in the different lineages of hMPXV (Clade IIb-A, A.2, A.1, A.1.1, and B.1). **(A)** Nucleotide and amino acid mutations in the Clade IIb-A lineages of viruses (n = 8) compared with NC_063383.1/ MT903340.1. **(B)** Nucleotide and amino acid mutationsFIGURE 2 (Continued) in the Clade IIb-A.2 lineages of viruses (*n* = 3) compared with NC_063383.1/ MT903340.1. **(C)** Nucleotide and amino acid mutations in the Clade IIb-A.1 lineages of viruses (*n* = 6) compared with NC_063383.1/ MT903340.1. **(D)** Nucleotide and amino acid mutations in the Clade IIb-A.1.1 lineages of viruses (n = 1) compared with NC_063383.1/ MT903340.1. **(E)** Nucleotide and amino acid mutations in the Clade IIb-B.1lineages of viruses (*n* = 963) compared with NC_063383.1/ MT903340.1.

Meanwhile, double-stranded DNA virus MPXVs are slow-evolving but have been reported to be susceptible to microevolution (amino acid point mutations) for human adaptation (Isidro et al., 2022). In this context, we are interested to find out the microevolution (amino acid point mutations) of the hMPXV-1A virus over different periods from 2017–2018 to 2022 (Isidro et al., 2022). For this purpose, in the present study, microevolution (amino acid point mutations) within and between lineages of hMPXV-1A, A.2, A.1, A.1.1, and B.1 lineages in clade-IIb viruses was measured using NCBI Reference Sequence NC_063383.1 in hMPXV-1A group as a reference sequence. Among these, NC_063383.1 and MT903340.1 were related to the isolate MPXV-M5312_HM12_Rivers, which showed the closest relationship in the phylogenetic analysis, and it was noteworthy that there was no difference in protein amino acid mutation level between them. The hMPXV-1A lineages of viruses compared to NC_063383.1/MT903340.1 virus exhibited nucleotide mutations in 44 proteins, of which 31 were non-synonymous, and 13 were synonymous (Figure 2A). The presence of more non-synonymous mutations in hMPXV-1A viruses suggests that these mutations may have arisen for host adaptations. Also, MPXVgp021: L124S and MPXVgp103: K606E amino acid mutations are present in high frequency among hMPXV-1A viruses (Figure 2A). From these, it appears that hMPXV-1A viruses undergo random multidirectional microevolution. Since MPXV is a zoonotic virus that can infect different hosts such as humans, monkeys, rodents, etc., it has been speculated that these viruses have undergone microevolution to adapt to the host by alternately infecting humans and animals in the 2017–2018 outbreak.

Similarly, A.2 viruses exhibited nucleotide mutations in 58 proteins compared to NC_063383.1/MT903340.1 virus, of which 33 mutations were non-synonymous, and 25 mutations were synonymous (Figure 2B). Of these 33 non-synonymous mutations, only 13 mutations are common among viruses in the A.2 group, and these 13 mutations include two mutations that are common among viruses in the hMPXV-1A (Figure 2B). Although hMPXV-1A and A.2 viruses randomly acquire mutations in many proteins, MPXVgp021: L124S and MPXVgp103: K606E mutations are conserved among these viruses (Figures 2A,B), so these could be considered as positive selection mutations.

Further, we are interested in finding mutations among viruses in group A.1. This analysis revealed mutations in 57 proteins in group A.1 viruses compared to NC_063383.1/MT903340.1 viruses (Figure 2C). Out of these 57 mutations, 29 are non-synonymous, and 28 are synonymous (Figure 2C). Of the 29 non-synonymous mutations, 10 are common among viruses in group A.1 (Figure 2C). It is also noteworthy that among these 10 amino acid mutations, MPXVgp021: L124S and MPXVgp103: K606E mutations are common in hMPXV-1A and A.2 groups (Figures 2A–C). Interestingly, it is noteworthy that the MT903344.1/MPXV-United Kingdom-P2 virus carries only 10 amino acid mutations that are common among viruses in the A.1 group (Supplementary Figure S1). Also, the MT903344.1/MPXV-United Kingdom-P2 virus must be used as a reference sequence among non-endemic MPXV viruses to measure mutations and evolution of B.1 viruses (Isidro et al., 2022). Next, we were interested in identifying mutations in the A.1.1 virus (MPXV/United States/2021/MD), the closest evolutionary ancestor of B.1 viruses (Figures 1A,B). Compared to NC_063383.1/MT903340.1 virus, A.1.1 (MPXV/United States/2021/MD) virus has mutations in 39 proteins, and of these 39 mutations, 26 are non-synonymous, and 13 are synonymous (Figure 2D). Among these 26 proteins amino acid mutations, 10 are in the MT903344.1/MPXV-United Kingdom-P2 or A.1 group of viruses (Figures 2C,D; Supplementary Figure S1). Excitingly, of the amino acid mutations in 26 proteins of the A.1.1 (MPXV/United States/2021/MD) virus, 22 were passed on to viruses in the B.1 group (Figures 2D,E). Further, mutations in group B.1 (compared with NC_063383.1/MT903340.1) are consistent with mutations found in a recent study comparing MT903344.1/MPXV-United Kingdom-P2 virus (Isidro et al., 2022).

**Figure 3:**
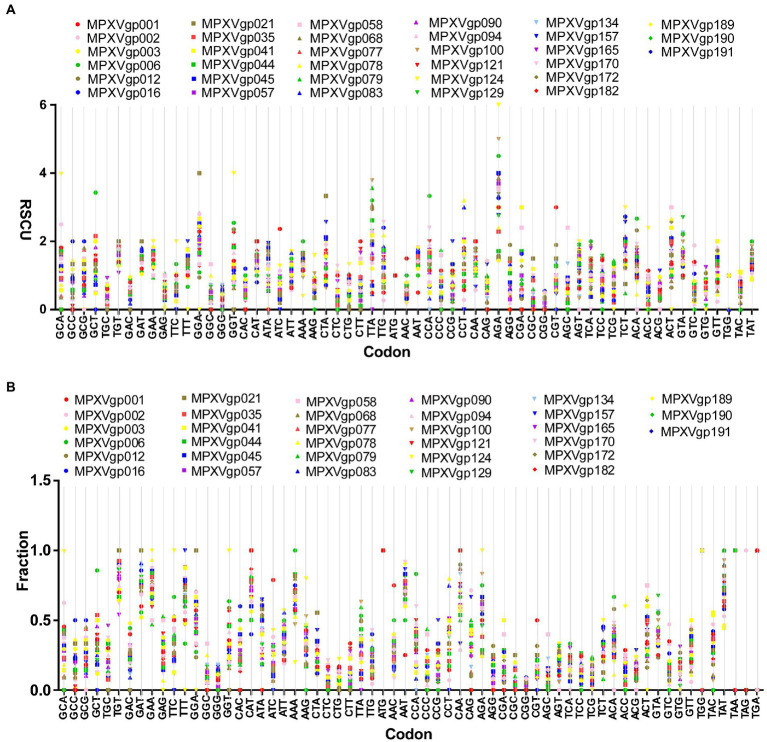
Relative Synonymous Codon Usage (RSCU) and Synonymous Codon Usage fraction. **(A)** Relative Synonymous Codon Usage (RSCU) for genes that displayed mutations in the Clade IIb-B.1 lineages of viruses compared with NC_063383.1/ MT903340.1. More than 1,000 sequences from different lineages in Clade IIb were used, and details are provided in Supplementary Data S1. The RSCU values of each gene are given in Supplementary Data S2. **(B)** Synonymous Codon Usage fraction for genes that displayed mutations in the Clade IIb-B.1 lineages of viruses compared with NC_063383.1/ MT903340.1. More than 1,000 sequences from different lineages in Clade IIb were used, and details are provided in Supplementary Data S1. The SCUF values of each gene are given in Supplementary Data S2.

**Figure 4:**
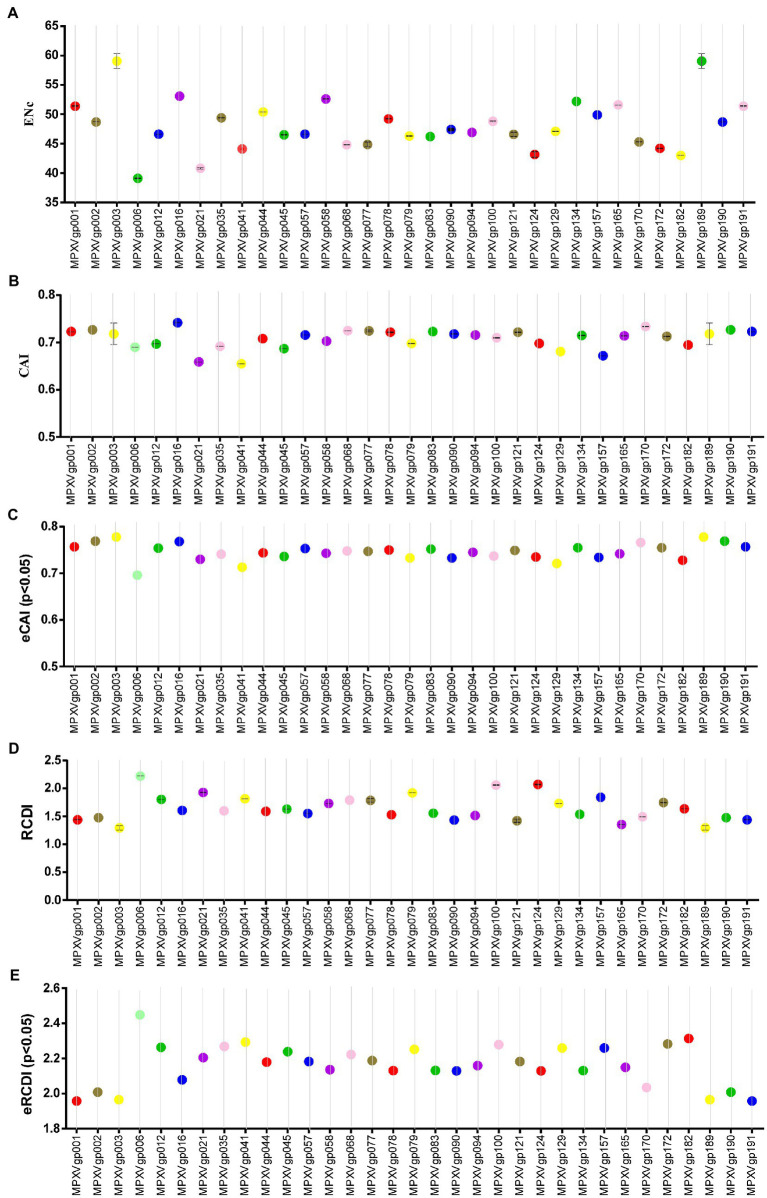
Host adaptation indices. **(A)** Effective Number of Codons (ENc) values for genes displayed mutations in the Clade IIb-B.1 lineages of viruses compared with NC_063383.1/ MT903340.1. More than 1,000 sequences from different lineages in Clade IIb were used, and details are provided in FIGURE 4 (Continued) FIGURE 4 (Continued) Supplementary Data S1. The ENc values of each gene are given in Supplementary Data S3. **(B)** Codon Adaptation Index (CAI) values for genes thatdisplayed nucleotide and amino acid mutations in the Clade IIb-B.1 lineages of viruses compared with NC_063383.1/ MT903340.1. More than 1,000 sequences from different lineages in Clade IIb were used, and details are provided in Supplementary Data S1. The CAI values of each gene are given in Supplementary Data S3. **(C)** Expected-CAI (eCAI) values for genes that displayed mutations in the Clade IIb-B.1 lineages of viruses compared with NC_063383.1/ MT903340.1. More than 1,000 sequences from different lineages in Clade IIb were used, and details are provided in Supplementary Data S1. The eCAI values of each gene are given in Supplementary Data S3. **(D)** Relative Codon Deoptimization Index (RCDI) values for genes that displayed mutations in the Clade IIb-B.1 lineages of viruses compared with NC_063383.1/ MT903340.1. More than 1,000 sequences from different lineages in Clade IIb were used, and details are provided in Supplementary Data S1. The RCDI values of each gene are given in Supplementary Data S4. **(E)** The expected value for the RCDI (eRCDI) values for genes that displayed mutations in the Clade IIb-B.1 lineages of viruses compared with NC_063383.1/ MT903340.1. More than 1,000 sequences from different lineages in Clade IIb were used, and details are provided in Supplementary Data S1. The eRCDI values of each gene are given in Supplementary Data S4.

**Figure 5:**
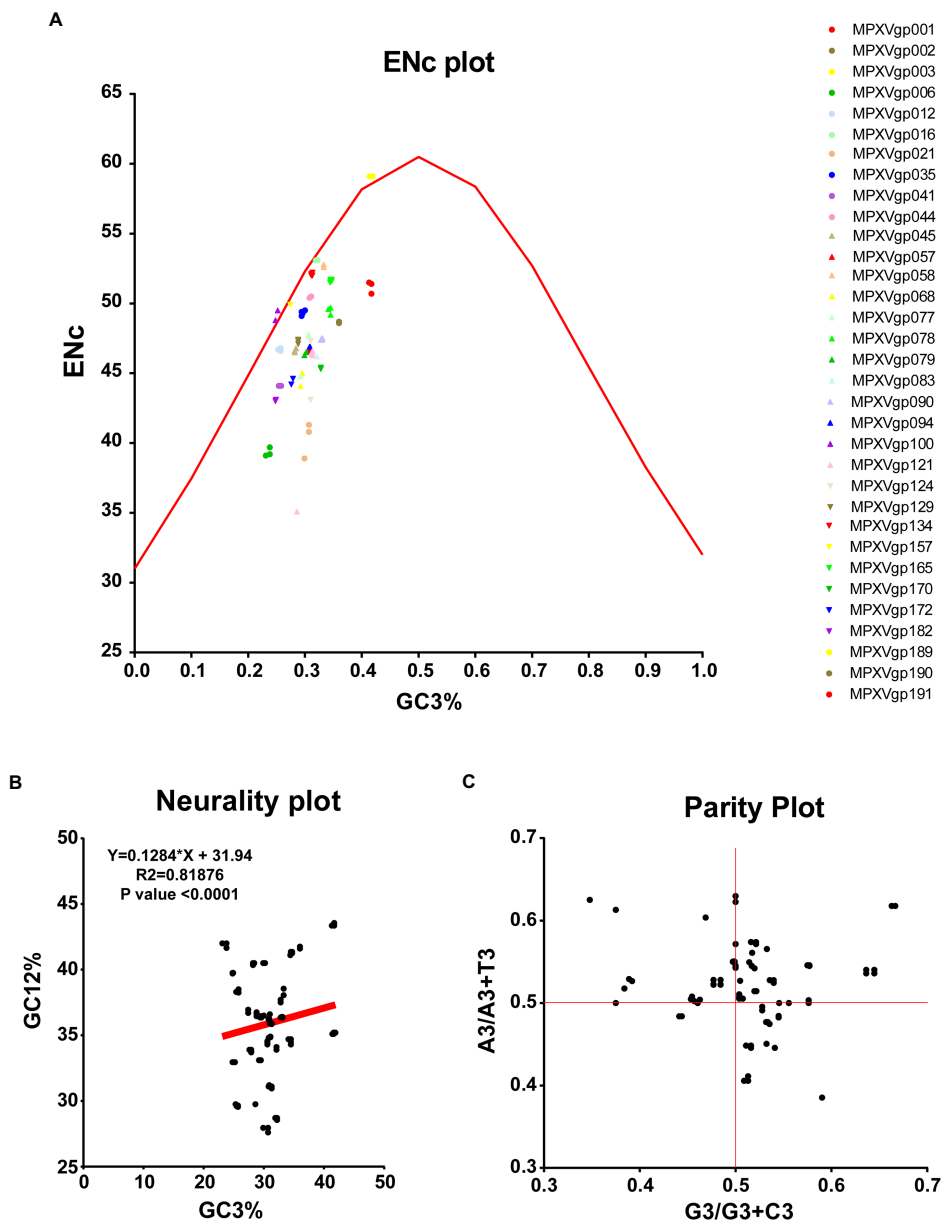
Selection and mutation pressure determining indices. **(A)** ENc-GC3s plot values for genes that displayed mutations in the Clade IIb-B.1lineages of viruses compared with NC_063383.1/ MT903340.1. More than 1,000 sequences from different lineages in Clade IIb were used, and details are provided in Supplementary Data S1. The ENc-GC3 values of each gene are given in Supplementary Data S3. **(B)** Neutrality plot values for genes that displayed mutations in the Clade IIb-B.1lineages of viruses compared with NC_063383.1/ MT903340.1. More than 1,000 sequences from different lineages in Clade IIb were used, and details are provided in Supplementary Data S1. The Neutrality plot values of each gene are given in Supplementary Data S3. **(C)** Parity Rule 2 (PR2)-bias plot values for genes that displayed mutations in the Clade IIb-B.1lineages of viruses compared with NC_063383.1/ MT903340.1. More than 1,000 sequences from different lineages in Clade IIb were used, and details are provided in Supplementary Data S1. The PR2-bias values of each gene are provided in Supplementary Data S5.

As B.1 lineages exhibited only point mutations in genes compared to NC_063383.1/MT903340.1, we were interested in determining the role of codon usage bias, host adaptation, selection pressure, and mutation pressure in the evolution of genes with these mutations. For this, we first performed RSCU, SCUF, and ENc analyses on genes that exhibited nucleotide mutations in lineage B.1 to detect codon usage bias. A RSCU value of 1 indicates no codon bias for that specific codon, >1.0 indicates positive codon usage bias (defined as abundant codons), and < 1.0 indicates negative codon usage bias (defined as less-abundant codons) (Gun et al., 2018; Wang et al., 2018). RSCU analysis revealed that the majority of codons for most genes were > 1.0 and < 1.0, indicating codon usage bias in these genes (Figure 3A). Similarly, SCUF analysis revealed bias in Synonymous Codon Usage Fraction in most genes (Figure 3B). As with the RSCU and SCUF analyses, most genes have ENc values of 40–50 (Figure 4A), indicating moderate codon usage bias in these genes. After this, we conducted CAI, eCAI, RCDI, and eRCDI analysis with human codon usage to find out whether the codon usage bias in these genes is likely to favor host (human) adaptation. In these analyses, genes with mutations in B.1 lineage showed CAI value of 0.65–0.75 (Figure 4B), eCAI value of 0.7–0.8 (Figure 4C), RCDI value of 1.3–2.2 (Figure 4D), and eRCDI value of 1.9–2.4 (Figure 4E). From the results of these analyses, it appears that codon usage in these genes is favorable for human adaptation. After this, we were interested to find out whether selection pressure or mutation pressure plays an important role in the evolution of these genes, which are favorable for human adaptation; for this, we carried out ENc-GC3s plot, Neutrality plot, and Parity Rule 2 (PR2)-bias plot analysis. In this Enc-plot analysis, since the values of most of the genes fall below the expected curve (Figure 5A), it is revealed that selection pressure plays a major role in the evolutionary development of these genes than mutation pressure. Similarly, the slope of the Neutrality plot analysis is 0.1284 (Y = 0.1284*X + 31.94, R^2^ = 0.81876, *p* value <0.0001), indicating that mutation pressure and natural selection pressure contribute 12.84 and 87.16%, respectively, in the evolution of these genes (Figure 5B). Finally, Parity Rule 2 (PR2)-bias plot analysis shows that most genes with mutations in B.1 lineage have unequal A to T and G to C (Figure 5C), which reveals the mutation and selection pressure in these genes. Collectively, the results of these analyzes show that there is a codon usage bias in the genes with mutations in the B.1 lineage, that the codon usage of these genes is favorable to human adaptation, and that selection pressure plays a major role in the evolution of these genes than mutation pressure.

More specifically, the mutations in the 5′-inverted terminal repeats (5’-ITR) [MPXVgp001 (Chemokine binding protein): S105L; MPXVgp002 (TNF receptor/CrmB): S54F; and MPXVgp003 (Ankyrin/Cop-C19L/J3L): D264N] and 3’-ITR [MPXVgp191 (Chemokine binding protein): S105L; MPXVgp190 (TNF receptor/CrmB): S54F; and MPXVgp189 (Ankyrin/Cop-C19L)/J1R): D264N] regions of viruses in group B.1 are identical (Supplementary Table S1), suggesting that these mutations are more likely to be functional mutations rather than random. Also, it is worth noting that the amino acid mutations that have gradually accumulated from 2017 to 2018 (hMPXV-1A) through A.1 and A.1.1 viruses to 2022 (B.1) are mostly in host immune evasion and virus propagation proteins (Supplementary Table S1). Furthermore, phylogenetic analysis, NBGMD analysis, and protein mutations suggest that A.2 viruses detected in the United States in 2021 and 2022 evolved directly from hMPXV-1A (2017–18) viruses and that A.2 viruses did not play a major role in the evolution of B.1 viruses through A.1 and A.1.1 viruses. Further, the MPXV/United States/2021/MD virus detected in 2021 in the United States is an evolutionary intermediate to the viruses responsible for the outbreak-2017–2018 in West Africa and B.1 viruses in phylogenetic, NBGMD and amino acid mutation levels. Particularly, since the data of complete genome sequences of MPXV viruses are not available in other countries especially in MPXV endemic countries after 2017–18, therefore it seems that these viruses have achieved evolution in MPXV non-endemic countries (United States, United Kingdom, Singapore, and Israel).

In conclusion, according to the available sequence data, MPXV viruses appear to have microevolved from 2017–18 to 2022, gradually accumulating mutations in key proteins to accommodate a multi-country outbreak-2022. Furthermore, different mutations in different proteins among individual viruses in the Clade IIb suggest that these mutations may have evolved as host adaptations. Since Poxviruses are slow evolving viruses, by monitoring MPXV viruses in other countries and animals and detecting unique mutations in MPXV viruses in different countries and animals, we can predict the geographical area/animals from which these viruses are spreading and determine the future direction of the outbreak, prevent disease transmission from different animals and countries, and is expected to support preparedness for future outbreaks.

## Materials and methods

### Data curation and sequence alignment

The MPXV’s whole genome sequences were retrieved from NCBI (National Center for Biotechnology Information)^1^ databases in the present study. Next, these MPXV’s whole genome sequences were aligned with MAFFT 7.407_1 multiple alignment program with parameters-Gap extend penalty (0.123) and Gap opening penalty (1.53) (Katoh and Standley, 2013; Mareuil et al., 2017; Lemoine et al., 2019).

### Phylogenetic analysis

For phylogenetic analysis, whole genome sequences of MPXVs aligned to MAFFT 7.407_1 determined the best fit model through the IQ-TREE web server^2^ (Trifinopoulos et al., 2016), and the GTR model was selected accordingly. Phylogenetic analysis of the whole genome of MPXVs was performed using PhyML 3.3_1 (Galaxy Version 3.3_1)/ngphylogeny.fr (Guindon et al., 2010; Mareuil et al., 2017; Lemoine et al., 2018), through GTR (Evolutionary model), discrete gamma model through categories (n = 4), Empirical (Equilibrium Frequencies), Subtree Pruning and Regraphing by tree topology search with optimizing parameters such as tree topology, branch length, and model parameter, and examination the branch support was performed out by approximate Bayes branch. Lastly, the interactive tree of life (iTOL) v5 (Letunic and Bork, 2021) was used to visualize the phylogenetic trees.

### Net between group mean distance analysis

In this study, the complete genome nucleotide sequences of different clades and lineages of MPXVs were first aligned in MAFFT 7.407_1 (Katoh and Standley, 2013; Mareuil et al., 2017; Lemoine et al., 2019; Desingu and Nagarajan, 2022a), and then NBGMD analysis was performed in MEGA7 (Kumar et al., 2016; Desingu and Nagarajan, 2022a). NBGMD analysis was executed in MEGA7 with the following parameters: Kimura 2-parameter model, Transitions plus Transversions substitution, gamma distribution-shape parameter = 5, gaps/missing data were pairwise deleted, and standard errors (assessed through the bootstrap test of 1,000 replicates). Finally, the measured standard errors were presented above the diagonal in the respective tables of NBGMD analysis.

### Identification mutations in the monkeypox virus

In the present study, Codon and amino acid mutations in hMPXV-A, A.2, A.1, A.1.1, and B.1 lineages were identified using MONKEYPOX VIRUS TYPING TOOL,^3^ NC_063383.1/MT903340.1 was used as a reference sequence.

### Relative synonymous codon usage analysis

RSCU value is measured as a ratio between the observed to the expected value of synonymous codons for a specified amino acid in the particular gene (Gun et al., 2018; Wang et al., 2018; Desingu and Nagarajan, 2022b). A RSCU value of 1 indicates no codon bias for that specific codon, >1.0 indicates positive codon usage bias (defined as abundant codons), and < 1.0 indicates negative codon usage bias (defined as less-abundant codons) (Gun et al., 2018; Wang et al., 2018). In this study, the RSCU values for different genes of MPXVs were measured using the ACUA Software (Vetrivel et al., 2007), and the details of the genes used and the number of sequences is shown in the respective figures.

### Effective number of codons

Effective number of codons determines which of the 61 codons are effectively used to make the 20 amino acids; this value ranges from 20 to 61. ENc values less than 35 indicate high codon bias, whereas ENc values greater than 50 indicate general random codon usage (Zhao et al., 2016; Wang et al., 2018; Desingu et al., 2022; Desingu and Nagarajan, 2022b). In this study, the ENc values of different genes of MPXV were determined through an online server^4^ (Puigbo et al., 2008a), and the details of the genes used and the number of sequences are shown in the respective figures.

### Codon adaptation index

The CAI is a quantitative measure of the resemblance of a given gene’s synonymous codon usage bias and the synonymous codon usage frequencies of a reference host gene (Puigbo et al., 2008a,b; Tian et al., 2020). The CAI values range from 0 to 1; when the CAI value is close to 1, it indicates a strong bias of the codon usage/protein expression (Puigbo et al., 2008a; Tian et al., 2020). The coding region nucleotide sequences of MPXV genes were retrieved from NCBI nucleotide public database (see text footnote 1). Thereference set of human (*Homo sapiens*) codon usage was obtained from the codon usage database.^5^ Then, the CAI was calculated using the online server (see footnote 4) (Puigbo et al., 2008a), and the details of the genes used and the number of sequences are shown in the respective figures.

### Expected-CAI

To determine if the statistically significant variations in the CAI values arise from codon usage bias, it is recommended to use expected-CAI (eCAI; Puigbo et al., 2008b; Cristina et al., 2015). We used the same coding nucleotide sequences of MPXV genes, and a reference set of host codon usage (human- *Homo sapiens*) was used in the CAI calculation, and the details of the genes used and the number of sequences is shown in the respective figures. The eCAI was measured through an online server^6^ at 95% confidence interval, using Markov method (Puigbo et al., 2008b; Cristina et al., 2015).

### Relative codon deoptimization index and expected value for the relative codon deoptimization index

The RCDI was introduced by Mueller (Mueller et al., 2006), measuring the codon deoptimization through the similarities in codon usage by the given genes (pathogen) and reference genomes (host- *Homo sapiens*) (Puigbo et al., 2010). When the RCDI value is close to 1, it indicates a higher rate of pathogen protein expression/adaptation in the host. The statistically significant difference in RCDI values was measured by eRCDI (Puigbo et al., 2010). The same data input used in CAI/eCAI was also utilized for the RCDI and eRCDI analysis also, and the details of the genes used and the number of sequences are shown in the respective figures. RCDI and eRCDI were measured on an online server,^7^ using Markov method at the 95% confidence interval (Puigbo et al., 2010).

### ENc-GC3s plot

In ENc-GC3s plot analysis, selection pressures or mutation pressures are the major factors affecting the codon usage bias of a particular gene is determined by plotting ENc values against the third position of GC3s of codon values (Tian et al., 2020; Desingu et al., 2022; Desingu and Nagarajan, 2022b). First, the expected curve was generated by assessing the expected ENc values for each GC3s as previously recommended (Wang et al., 2018; Tian et al., 2020; Desingu et al., 2022). The ENc value and GC3s value for different genes MPXVs were calculated through an online server for CAI calculation (see footnote 4) (Puigbo et al., 2008a). When mutation pressure alone determines the codon usage bias of a particular gene, the ENc-GC3s plot value lies on or around the expected curve, but when codon usage bias is influenced by selection and other factors, the ENc-GC3s plot value falls significantly below the expected curve (Wang et al., 2018; Tian et al., 2020).

### Neutrality plot analysis

The degree of influence of mutation pressure and natural selection on the codon usage patterns is evaluated by GC12 values of the codon plotted against GC3 values in the neutrality plot analysis (Desingu et al., 2022; Desingu and Nagarajan, 2022b). The GC12 and GC3 values for the nucleotide sequences of different genes of MPXVs were obtained from an online CAI analysis server (see footnote 4) (Puigbo et al., 2008a).

### Parity rule 2-bias plot

In PR2-bias plot analysis, the mutation pressure and natural selection affecting the codon usage bias is measured by the AT bias [A3/(A3+ T3)] is plotted against GC-bias [G3/(G3 + C3)] (Tian et al., 2020; Desingu et al., 2022; Desingu and Nagarajan, 2022b). Parity Rule 2 (PR2)-bias plot analysis shows that mutation and natural selection pressure are absent if A = T and G = C (Tian et al., 2020; Desingu et al., 2022; Desingu and Nagarajan, 2022b). The A3, T3, G3, and C3 values of nucleotide sequences of different genes of MPXVs were attained by using the ACUA Software (Vetrivel et al., 2007).

## Data availability statement

The original contributions presented in the study are included in the article/Supplementary material, further inquiries can be directed to the corresponding author.

## Author contributions

PD analyzed and wrote the first draft of the manuscript. TR reviewed the manuscript. All authors contributed significantly to this manuscript and reviewed and approved the final submission.

## Funding

PD is a DST-INSPIRE Faculty supported by research funding from the Department of Science and Technology, India (DST/INSPIRE/04/2016/001067), and Science and Engineering Research Board, Department of Science and Technology, India (CRG/2018/002192).

## Conflict of interest

The authors declare that the research was conducted in the absence of any commercial or financial relationships that could be construed as a potential conflict of interest.

## Publisher’s note

All claims expressed in this article are solely those of the authors and do not necessarily represent those of their affiliated organizations, or those of the publisher, the editors and the reviewers. Any product that may be evaluated in this article, or claim that may be made by its manufacturer, is not guaranteed or endorsed by the publisher.

## Supplementary material

The Supplementary material for this article can be found online at: https://www.frontiersin.org/articles/10.3389/fmicb.2022.1037598/full#supplementary-material

## Footnotes

^1^ https://www.ncbi.nlm.nih.gov/nucleotide/

^2^ http://iqtree.Cibiv.univie.ac.at/

^3^ https://www.genomedetective.com/app/typingtool/monkeypox/

^4^ http://ppuigbo.me/programs/CAIcal/

^5^ http://ppuigbo.me/programs/CAIcal/CU_human_nature

^6^ http://ppuigbo.me/programs/CAIcal/E-CAI/calc_random.php

^7^ http://ppuigbo.me/programs/CAIcal//RCDI/

## References

[ref1] Adler H. Gould S. Hine P. Snell L. B. Wong W. Houlihan C. F.. (2022). Clinical features and management of human monkeypox: a retrospective observational study in the United Kingdom. Lancet Infect. Dis. 22, 1153–1162. doi: 10.1016/S1473-3099(22)00228-6, PMID: 35623380 PMC9300470

[ref2] Breman J. G. Kalisa-Ruti Steniowski M. V. Zanotto E. Gromyko A. I. Arita I. (1980). Human monkeypox, 1970–1979. Bull. World Health Organ. 58, 165–182. 6249508 PMC2395797

[ref3] Brown K. Leggat P. A. (2016). Human Monkeypox: current state of knowledge and implications for the future. Trop. Med. Infect. Dis. 1:8. doi: 10.3390/tropicalmed1010008, PMID: 30270859 PMC6082047

[ref4] Costello V. Sowash M. Gaur A. Cardis M. Pasieka H. Wortmann G.. (2022). Imported monkeypox from international traveler, Maryland, United States, 2021. Emerg. Infect. Dis. 28, 1002–1005. doi: 10.3201/eid2805.220292, PMID: 35263559 PMC9045429

[ref5] Cristina J. Moreno P. Moratorio G. Musto H. (2015). Genome-wide analysis of codon usage bias in Ebolavirus. Virus Res. 196, 87–93. doi: 10.1016/j.virusres.2014.11.005, PMID: 25445348

[ref6] Desingu P. A. N. K. (2022). Genomic regions insertion and deletion in monkeypox virus causing multi-country outbreak-2022. bioRxiv. doi: 10.1101/2022.06.28.497936

[ref7] Desingu P. A. Nagarajan K. (2022a). Detection of beak and feather disease virus in India and its implications. Transbound. Emerg. Dis. doi: 10.1111/tbed.14749, PMID: 36316791

[ref8] Desingu P. A. Nagarajan K. (2022b). Genetic diversity and characterization of circular replication (rep)-encoding single-stranded (CRESS) DNA viruses. Microbiol Spectr. 8:e0105722. doi: 10.1128/spectrum.01057-22, PMID: 36346238 PMC9769708

[ref9] Desingu P. A. Nagarajan K. Dhama K. (2022). SARS-CoV-2 gained a novel spike protein S1-N-terminal domain (S1-NTD). Environ. Res. 211:113047. doi: 10.1016/j.envres.2022.113047, PMID: 35292244 PMC8917877

[ref11] Formenty P. Muntasir M. O. Damon I. Chowdhary V. Opoka M. L. Monimart C.. (2010). Human monkeypox outbreak caused by novel virus belonging to Congo Basin clade, Sudan, 2005. Emerg. Infect. Dis. 16, 1539–1545. doi: 10.3201/eid1610.100713, PMID: 20875278 PMC3294404

[ref12] Gispen R. Verlinde J. D. Zwart P. (1967). Histopathological and virological studies on monkeypox. Arch. Gesamte Virusforsch. 21, 205–216. doi: 10.1007/BF01241445 4298424

[ref13] Guindon S. Dufayard J. F. Lefort V. Anisimova M. Hordijk W. Gascuel O. (2010). New algorithms and methods to estimate maximum-likelihood phylogenies: assessing the performance of PhyML 3.0. Syst. Biol. 59, 307–321. doi: 10.1093/sysbio/syq010, PMID: 20525638

[ref14] Gun L. Yumiao R. Haixian P. Liang Z. (2018). Comprehensive analysis and comparison on the codon usage pattern of whole mycobacterium tuberculosis coding genome from different area. Biomed. Res. Int. 2018:3574976. doi: 10.1155/2018/3574976, PMID: 29854746 PMC5964552

[ref15] Happi C. Adetifa I. Mbala P. Njouom R. Nakoune E. Happi A.. (2022). Urgent need for a non-discriminatory and non-stigmatizing nomenclature for monkeypox virus. PLoS Biol. 20:e3001769. doi: 10.1371/journal.pbio.3001769, PMID: 35998195 PMC9451062

[ref16] Heymann D. L. Szczeniowski M. Esteves K. (1998). Re-emergence of monkeypox in Africa: a review of the past six years. Br. Med. Bull. 54, 693–702. doi: 10.1093/oxfordjournals.bmb.a011720, PMID: 10326294

[ref17] Hutin Y. J. Williams R. J. Malfait P. Pebody R. Loparev V. N. Ropp S. L.. (2001). Outbreak of human monkeypox, Democratic Republic of Congo, 1996 to 1997. Emerg. Infect. Dis. 7, 434–438. doi: 10.3201/eid0703.010311, PMID: 11384521 PMC2631782

[ref18] Isidro J. Borges V. Pinto M. Sobral D. Santos J. D. Nunes A.. (2022). Phylogenomic characterization and signs of microevolution in the 2022 multi-country outbreak of monkeypox virus. Nat. Med. 28, 1569–1572. doi: 10.1038/s41591-022-01907-y, PMID: 35750157 PMC9388373

[ref19] Jezek Z. Marennikova S. S. Mutumbo M. Nakano J. H. Paluku K. M. Szczeniowski M. (1986). Human monkeypox: a study of 2,510 contacts of 214 patients. J. Infect. Dis. 154, 551–555. doi: 10.1093/infdis/154.4.551, PMID: 3018091

[ref20] Katoh K. Standley D. M. (2013). MAFFT multiple sequence alignment software version 7: improvements in performance and usability. Mol. Biol. Evol. 30, 772–780. doi: 10.1093/molbev/mst010, PMID: 23329690 PMC3603318

[ref21] Kugelman J. R. Johnston S. C. Mulembakani P. M. Kisalu N. Lee M. S. Koroleva G.. (2014). Genomic variability of monkeypox virus among humans, Democratic Republic of the Congo. Emerg. Infect. Dis. 20, 232–239. doi: 10.3201/eid2002.130118, PMID: 24457084 PMC3901482

[ref22] Kumar S. Stecher G. Tamura K. (2016). MEGA7: molecular evolutionary genetics analysis version 7.0 for bigger datasets. Mol. Biol. Evol. 33, 1870–1874. doi: 10.1093/molbev/msw054, PMID: 27004904 PMC8210823

[ref23] Ladnyj I. D. Ziegler P. Kima E. (1972). A human infection caused by monkeypox virus in Basankusu territory, Democratic Republic of the Congo. Bull. World Health Organ. 46, 593–597. 4340218 PMC2480792

[ref24] Learned L. A. Reynolds M. G. Wassa D. W. Li Y. Olson V. A. Karem K.. (2005). Extended interhuman transmission of monkeypox in a hospital community in the Republic of the Congo, 2003. Am. J. Trop. Med. Hyg. 73, 428–434. doi: 10.4269/ajtmh.2005.73.428 16103616

[ref25] Lemoine F. Domelevo Entfellner J. B. Wilkinson E. Correia D. Dávila Felipe M. De Oliveira T.. (2018). Renewing Felsenstein’s phylogenetic bootstrap in the era of big data. Nature 556, 452–456. doi: 10.1038/s41586-018-0043-0, PMID: 29670290 PMC6030568

[ref26] Lemoine F. Correia D. Lefort V. Doppelt-Azeroual O. Mareuil F. Cohen-Boulakia S.. (2019). NGPhylogeny.Fr: new generation phylogenetic services for non-specialists. Nucleic Acids Res. 47, W260–W265. doi: 10.1093/nar/gkz303, PMID: 31028399 PMC6602494

[ref27] Letunic I. Bork P. (2021). Interactive tree of life (iTOL) v5: an online tool for phylogenetic tree display and annotation. Nucleic Acids Res. 49, W293–W296. doi: 10.1093/nar/gkab301, PMID: 33885785 PMC8265157

[ref10] Mareuil F. Doppelt-Azeroual O. Ménager H. A. (2017). public Galaxy platform at Pasteur used as an execution engine for web services. F1000Research 6:1030. doi: 10.7490/f1000research.1114334.1

[ref28] Mauldin M. R. McCollum A. M. Nakazawa Y. J. Mandra A. Whitehouse E. R. Davidson W.. (2022). Exportation of Monkeypox virus from the African continent. J. Infect. Dis. 225, 1367–1376. doi: 10.1093/infdis/jiaa559, PMID: 32880628 PMC9016419

[ref29] Mcconnell S. J. H. Y. Mattson D. E. Erickson L. (1962). Monkey pox disease in irradiated cynomologous monkeys. Nature 195, 1128–1129. doi: 10.1038/1951128a0

[ref30] Mueller S. Papamichail D. Coleman J. R. Skiena S. Wimmer E. (2006). Reduction of the rate of poliovirus protein synthesis through large-scale codon deoptimization causes attenuation of viral virulence by lowering specific infectivity. J. Virol. 80, 9687–9696. doi: 10.1128/JVI.00738-06, PMID: 16973573 PMC1617239

[ref31] Muhlemann B. Vinner L. Margaryan A. Wilhelmson H. de la Fuente Castro C. Allentoft M. E.. (2020). Diverse variola virus (smallpox) strains were widespread in northern Europe in the Viking age. Science 369:eaaw8977. doi: 10.1126/science.aaw8977, PMID: 32703849

[ref32] Ng O. T. Lee V. Marimuthu K. Vasoo S. Chan G. Lin R. T. P.. (2019). A case of imported Monkeypox in Singapore. Lancet Infect. Dis. 19:1166. doi: 10.1016/S1473-3099(19)30537-7, PMID: 31657773 PMC7129797

[ref33] Nolen L. D. Osadebe L. Katomba J. Likofata J. Mukadi D. Monroe B.. (2016). Extended human-to-human transmission during a Monkeypox outbreak in the Democratic Republic of the Congo. Emerg. Infect. Dis. 22, 1014–1021. doi: 10.3201/eid2206.150579, PMID: 27191380 PMC4880088

[ref34] Parker S. Buller R. M. (2013). A review of experimental and natural infections of animals with monkeypox virus between 1958 and 2012. Future Virol. 8, 129–157. doi: 10.2217/fvl.12.130, PMID: 23626656 PMC3635111

[ref35] Perumal Arumugam Desingu K. N. (2022). Genomic regions insertion and deletion in monkeypox virus causing multi-country outbreak-2022. bioRxiv. doi: 10.1101/2022.06.28.497936

[ref36] Puigbo P. Aragones L. Garcia-Vallve S. (2010). RCDI/eRCDI: a web-server to estimate codon usage deoptimization. BMC. Res. Notes 3:87. doi: 10.1186/1756-0500-3-87, PMID: 20356391 PMC2853550

[ref37] Puigbo P. Bravo I. G. Garcia-Vallve S. (2008a). CAIcal: a combined set of tools to assess codon usage adaptation. Biol. Direct 3:38. doi: 10.1186/1745-6150-3-38, PMID: 18796141 PMC2553769

[ref38] Puigbo P. Bravo I. G. Garcia-Vallve S. (2008b). E-CAI: a novel server to estimate an expected value of codon adaptation index (eCAI). BMC Bioinformatics 9:65. doi: 10.1186/1471-2105-9-65, PMID: 18230160 PMC2246156

[ref39] Reynolds M. G. Damon I. K. (2012). Outbreaks of human monkeypox after cessation of smallpox vaccination. Trends Microbiol. 20, 80–87. doi: 10.1016/j.tim.2011.12.001, PMID: 22239910

[ref40] Reynolds M. G. Davidson W. B. Curns A. T. Conover C. S. Huhn G. Davis J. P.. (2007). Spectrum of infection and risk factors for human monkeypox, United States, 2003. Emerg. Infect. Dis. 13, 1332–1339. doi: 10.3201/eid1309.070175, PMID: 18252104 PMC2857287

[ref41] Rezza G. (2019). Emergence of human monkeypox in West Africa. Lancet Infect. Dis. 19, 797–799. doi: 10.1016/S1473-3099(19)30281-6 31285141

[ref42] Rimoin A. W. Mulembakani P. M. Johnston S. C. Lloyd Smith J. O. Kisalu N. K. Kinkela T. L.. (2010). Major increase in human monkeypox incidence 30 years after smallpox vaccination campaigns cease in the Democratic Republic of Congo. Proc. Natl. Acad. Sci. U. S. A. 107, 16262–16267. doi: 10.1073/pnas.1005769107, PMID: 20805472 PMC2941342

[ref43] Tian H. F. Hu Q. M. Xiao H. B. Zeng L. B. Meng Y. Li Z. (2020). Genetic and codon usage bias analyses of major capsid protein gene in Ranavirus. Infect. Genet. Evol. 84:104379. doi: 10.1016/j.meegid.2020.104379, PMID: 32497680

[ref44] Trifinopoulos J. Nguyen L. T. von Haeseler A. Minh B. Q. (2016). W-IQ-TREE: a fast online phylogenetic tool for maximum likelihood analysis. Nucleic Acids Res. 44, W232–W235. doi: 10.1093/nar/gkw256, PMID: 27084950 PMC4987875

[ref45] Vetrivel U. Arunkumar V. Dorairaj S. (2007). ACUA: a software tool for automated codon usage analysis. Bioinformation 2, 62–63. doi: 10.6026/97320630002062, PMID: 18188422 PMC2174420

[ref46] Von Magnus P. A. E. Petersen K. B. Birch-Andersen A. (1956). A pox-like disease in cynomolgus monkeys. Acta Pathol. Microbiol. Scand. 46, 156–176.

[ref47] Wang L. Xing H. Yuan Y. Wang X. Saeed M. Tao J.. (2018). Genome-wide analysis of codon usage bias in four sequenced cotton species. PLoS One 13:e0194372. doi: 10.1371/journal.pone.0194372, PMID: 29584741 PMC5870960

[ref48] Yinka-Ogunleye A. Aruna O. Dalhat M. Ogoina D. McCollum A. Disu Y.. (2019). Outbreak of human monkeypox in Nigeria in 2017-18: a clinical and epidemiological report. Lancet Infect. Dis. 19, 872–879. doi: 10.1016/S1473-3099(19)30294-4, PMID: 31285143 PMC9628943

[ref49] Zhao Y. Zheng H. Xu A. Yan D. Jiang Z. Qi Q.. (2016). Analysis of codon usage bias of envelope glycoprotein genes in nuclear polyhedrosis virus (NPV) and its relation to evolution. BMC Genomics 17:677. doi: 10.1186/s12864-016-3021-7, PMID: 27558469 PMC4997668

