# Supplementary material for: Evolution of monkeypox virus from 2017 to 2022: In the light of point mutations
Source: Front Microbiol. 2022 Dec 14;13:1037598. doi: 10.3389/fmicb.2022.1037598 (PMC9795006; doi:10.3389/fmicb.2022.1037598)
Supplement: Supplementary file 6 [file Table_6.DOCX]

| **S.No.** | **Proteins** | **Mutation** | **Note of the proteins** |
| --- | --- | --- | --- |
|  | MPXVgp001 (QNP13600.1) | S105L | Chemokine binding protein (Cop-C23L); J1L; similar to Vaccinia virus strain Copenhagen C23L |
|  | MPXVgp002 (QNP13601.1) | S54F | TNF receptor (CrmB) (Cop-C22L); J2L |
|  | MPXVgp003 (QNP13602.1) | D264N | Ankyrin (Cop-C19L); J3L |
|  | MPXVgp012 (QNP13609.1) | A423D | Ankyrin; Type I IFN resistance (Cop-C9L); D9L; similar to Vaccinia virus strain Copenhagen C9L; ankyrin-like |
|  | MPXVgp035 (QNP13628.1) | R48C | Kelch-like protein (Cop-F3L); C9L; similar to Vaccinia virus strain Copenhagen F3L; kelch-like |
|  | MPXVgp041 (QNP13634.1) | P78S | S-S bond formation pathway protein substrate (Cop-F9L); C15L; similar to Vaccinia virus strain Copenhagen F9L |
|  | MPXVgp044 (QNP13637.1) | E125K | EEV maturation protein (Cop-F12L); C18L; similar to Vaccinia virus strain Copenhagen F12L; actin tail formation |
|  | MPXVgp057 (QNP13652.1) | L108F | DNA polymerase (Cop-E9L); F8L; similar to Vaccinia virus strain Copenhagen E9L; DNA polymerase, catalytic subunit |
|  | MPXVgp058 (QNP13653.1) | D56N | Sulfhydryl oxidase (FAD-linked) (Cop-E10R); F9R;similar to Vaccinia virus strain Copenhagen E10R; protein disulfide bond-forming enzyme |
|  | MPXVgp077 (QNP13672.1) | D196N | Virion phosphoprotein, early morphogenesis (Cop-G7L); G8L; similar to Vaccinia virus strain Copenhagen G7L; virion protein |
|  | MPXVgp078 (QNP13673.1) | S30L, D88N | VLTF-1 (late transcription factor 1) (Cop-G8R); G9R; similar to Vaccinia virus strain Copenhagen G8R; late gene transcription factor, VLTF-1 |
|  | MPXVgp090 (QNP13685.1) | S734L | RNA polymerase subunit (RPO147) (Cop-J6R); L6R; similar to Vaccinia virus strain Copenhagen J6R; RNA polymerase, 147 kDa subunit |
|  | MPXVgp094 (QNP13689.1) | H740Y | RAP94 (RNA pol assoc protein) (Cop-H4L); H4L; similar to Vaccinia virus strain Copenhagen H4L; virion core RNA polymerase-associated protein, RAP94 |
|  | MPXVgp121 (QNP13716.1) | D98N | P4a precursor (Cop-A10L); A11L; similar to Vaccinia virus strain Copenhagen A10L; major virion core protein p4a |
|  | MPXVgp124 (QNP13719.1) | A17T | IMV membrane protein, virion maturation (Cop-A13L); A14L; similar to Vaccinia virus strain Copenhagen A13L; IMV inner and outer membrane protein |
|  | MPXVgp129 (QNP13725.1) | E62K, T129I, R243Q, E435K | DNA helicase, transcript release factor (Cop-A18R); A19R; similar to Vaccinia virus strain Copenhagen A18R; virion core associated DNA helicase; post-replicative negative transcription elongation factor |
|  | MPXVgp134 (QNP13730.1) | S307L | VITF-3 45kda subunit (Cop-A23R); A24R; similar toVaccinia virus strain Copenhagen A23R; intermediate transcription factor, VITF-3, 45 kDa |
|  | MPXVgp170 (QNP13763.1) | L263F | Soluble IFN-g receptor-like protein (Cop-B8R); B9R; similar to Vaccinia virus strain Copenhagen B8R; secreted IFN-g binding protein |
|  | MPXVgp182 (QNP13774.1) | D209N, P722S, M1741I | Surface glycoprotein; B21R; putative membrane-associated glycoprotein; cadherin-like domain |
|  | MPXVgp189 (QNP13778.1) | D264N | Ankyrin (Cop-C19L); J1R; ankyrin-like |
|  | MPXVgp190 (QNP13779.1) | S54F | TNF receptor (CrmB) (Cop-C22L); J2R; secreted TNF binding protein |
|  | MPXVgp191 (QNP13780.1) | S105L | Chemokine binding protein (Cop-C23L); J3R; similar to Vaccinia virus strain Copenhagen B29R; CC-chemokine binding |

**Supplementary Table 1:** Proteins notes are provided for the proteins harboring amino acid mutations in the Clade IIb-B.1 lineage. This proteins note is taken from the Genbank annotation (this description relies exclusively on the GenBank annotation)
