# Supplementary figures and images for: Evolution of monkeypox virus from 2017 to 2022: In the light of point mutations
Source: Front Microbiol. 2022 Dec 14;13:1037598. doi: 10.3389/fmicb.2022.1037598 (PMC9795006; doi:10.3389/fmicb.2022.1037598)

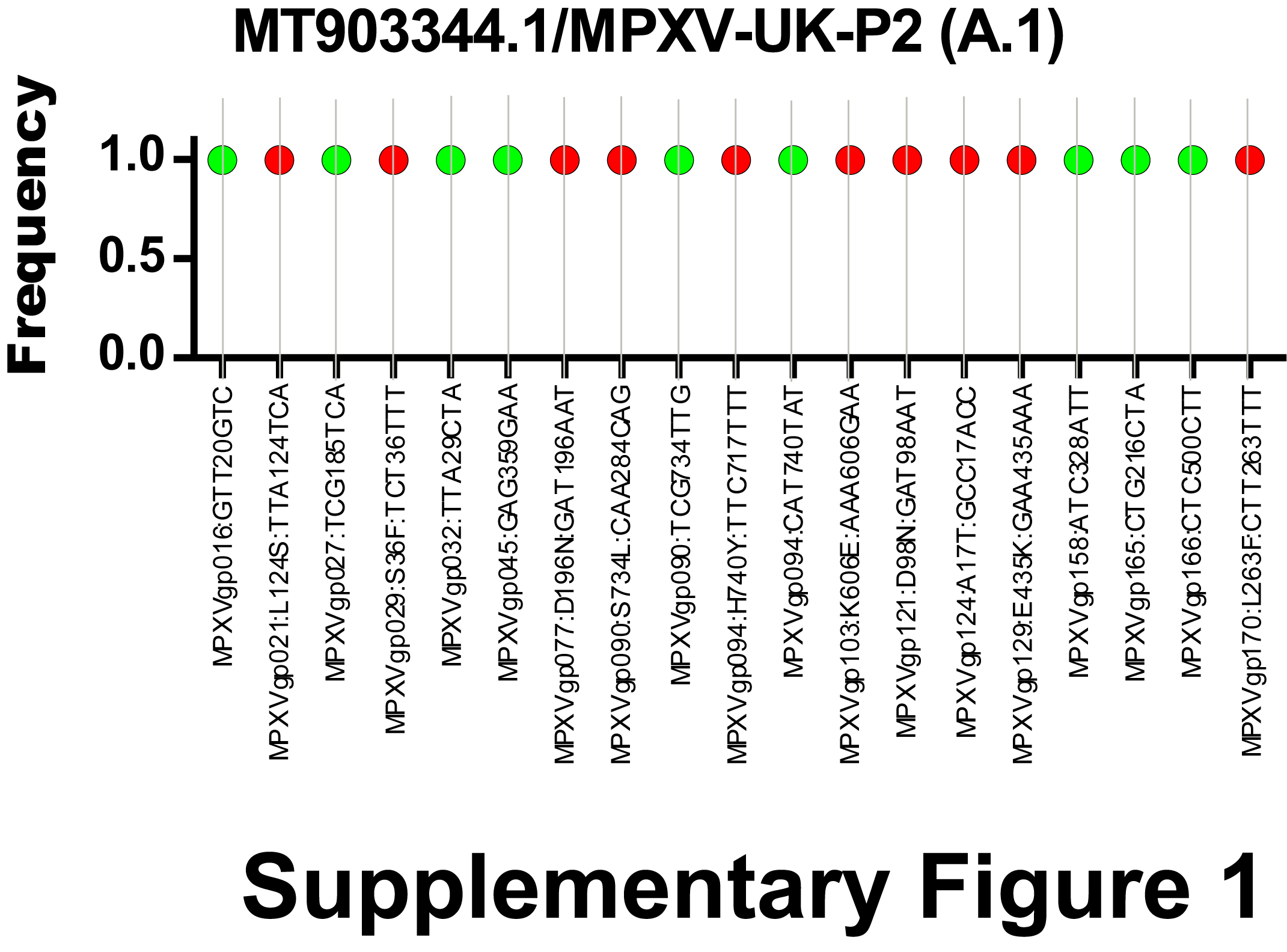

Supplement: Supplementary file 7 [file Image_1.TIF]
